# Retraction Note: Globalization and vulnerable populations in times of a pandemic: A Mayan perspective

**DOI:** 10.1186/s13010-020-00098-z

**Published:** 2020-12-14

**Authors:** Claudia Ruiz Sotomayor, Alejandra Barrero

**Affiliations:** 1 Pellegrino Center for Clinical Bioethics, Georgetown University, Washington, DC USA; 2 Department of Neonatology, Beth Israel Deaconess Medical Center, Boston, Massachusetts Harvard Medical School, Department of Pediatrics, Boston, Massachusetts USA

**Retraction Note: Philos Ethics Humanit Med (2020) 15:9**

**https://doi.org/10.1186/s13010-020-00093-4**

The Publisher has retracted this article [1] because an incorrect version was published in error. The correct version is published as [2]. Springer Nature apologises to the authors and to readers. All authors agree to this retraction.

[1] Sotomayor, C.R., Barrero, A. Globalization and vulnerable populations in times of a pandemic: A Mayan perspective. Philos Ethics Humanit Med 15, 9 (2020). 10.1186/s13010-020-00093-4

[2] Sotomayor, C.R., Barrero, A. Globalization and vulnerable populations in times of a pandemic: a Mayan perspective. Philos Ethics Humanit Med (2020). 10.1186/s13010-020-00097-0

## References

[1] Sotomayor CR, Barrero A (2020). Globalization and vulnerable populations in times of a pandemic: A Mayan perspective. Philos Ethics Humanit Med.

